# Glycyrrhizic Acid: A Natural Plant Ingredient as a Drug Candidate to Treat COVID-19

**DOI:** 10.3389/fphar.2021.707205

**Published:** 2021-07-09

**Authors:** Zhong Sun, Guozhong He, Ninghao Huang, Karuppiah Thilakavathy, Jonathan Chee Woei Lim, S. Suresh Kumar, Chenglong Xiong

**Affiliations:** ^1^ Department of Biomedical Science, Faculty of Medicine and Health Sciences, Universiti Putra Malaysia, Serdang, Malaysia; ^2^ School of Public Health, Kunming Medical University, Kunming, China; ^3^ Department of Epidemiology, School of Public Health, Fudan University, Shanghai, China; ^4^ Key Laboratory of Public Health Safety, Ministry of Education, Fudan University, Shanghai, China; ^5^ Genetics and Regenerative Medicine Research Group, Faculty of Medicine and Health Sciences, Universiti Putra Malaysia, Serdang, Malaysia; ^6^ Department of Medicine, Faculty of Medicine and Health Sciences, Universiti Putra Malaysia, Serdang, Malaysia; ^7^ Centre for Materials Engineering and Regenerative Medicine, Bharath Institute of Higher Education and Research, Chennai, India

**Keywords:** COVID-19, SARS-CoV-2, glycyrrhizic acid, glycyrrhizin, immune synergy, steroid metabolism

## Abstract

The total number of cumulative cases and deaths from the COVID-19 pandemic caused by SARS-CoV-2 is still increasing worldwide. Although many countries have actively implemented vaccination strategies to curb the epidemic, there is no specific efficient therapeutic drug for this virus to effectively reduce deaths. Therefore, the underappreciated macromolecular compounds have become the spotlight of research. Furthermore, the medicinal compounds in plants that provide myriad possibilities to treat human diseases have become of utmost importance. Experience indicates that Traditional Chinese medicine effectively treats SARS and has been used for treating patients with COVID-19 in China. As one of the world’s oldest herbal remedies, licorice is used for treating patients with all stages of COVID-19. Glycyrrhizic acid (GA), the main active compound in licorice, has been proven effective in killing the SARS virus. Meanwhile, as a natural plant molecule, GA can also directly target important protein structures of the SARS-CoV-2 virus and inhibit the replication of SARS-CoV-2. In this review, we summarized the immune synergy of GA and its potential role in treating COVID-19 complications. Besides, we reviewed its anti-inflammatory effects on the immune system and its positive effects in cooperation with various drugs to fight against COVID-19 and its comorbidities. The purpose of this review is to elucidate and suggest that GA can be used as a potential drug during COVID-19 treatment.

## Introduction

Coronavirus disease 2019 (COVID-19) pandemic is caused by severe acute respiratory syndrome coronavirus 2 (SARS-CoV-2) (Lai et al., 2020b). Based on previous experience in managing pandemics, a safe and effective vaccine could reduce virus transmission (Lipsitch and Dean, 2020). Therefore, several COVID-19 vaccines with high efficacy levels have been widely accepted worldwide, and most people are willing to get the vaccination (WHO, 2020a; Lazarus et al., 2021). However, the vaccines are not 100% effective (WHO, 2020a). As of May 9, 2021, 156 million cases of COVID-19 have been reported to the World Health Organization (WHO) in various countries worldwide, and more than 3.2 million people have died as a result (WHO, 2020b). Due to the lack of specific antiviral therapeutics, the primary treatment strategy for COVID-19 is supportive care, supplemented by broad-spectrum antiviral and antibiotics, drugs for preventing cytokine storm, corticosteroids as well as healing plasma from infected patients (Chen et al., 2020b). The corticosteroid drug dexamethasone has proven to improve patient's survival rates with severe COVID-19 (WHO, 2020c).

**FIGURE 1:**
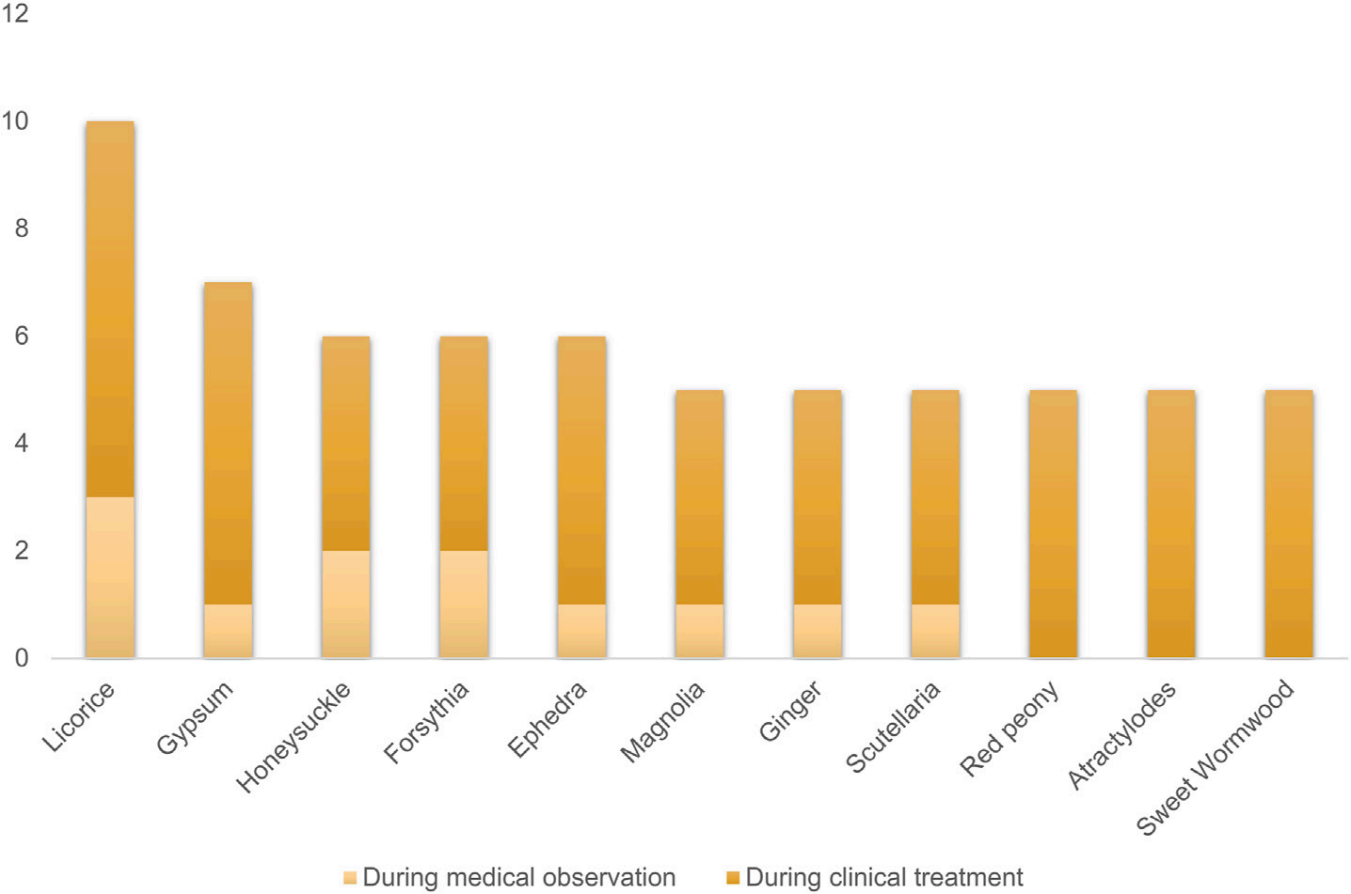
The top 10 most frequently used herbs among the recommended Chinese medicine formulas for COVID-19 treatment.

Interestingly, traditional Chinese medicine has been promoted as a treatment for COVID-19 in China and some other countries (Yang et al., 2020b; Cyranoski, 2020), although there is a lack of sufficient evidence. But in fact, statistical analysis has shown that integrated traditional Chinese medicine and Western medicine have effectively reduced the mortality rate during the SARS virus outbreak (Chen and Nakamura, 2004). According to the “Diagnosis and Treatment Protocol for Novel Coronavirus Pneumonia” issued by the National Health Commission of China (NHC) (Pei-Fang, 2020), licorice is the most frequently used herbal medicine among all recommended Chinese medicine formulas for COVID-19 treatment (Figure 1).

Licorice has a long history in traditional Chinese medicine, especially on its antiviral and antibacterial effects (Wang et al., 2015). The bibliometric analysis results show that glycyrrhizic acid (GA, also known as glycyrrhizin) is the most used molecule in licorice research for antiviral (Ram, 2015; Gomaa and Abdel-Wadood, 2021). As one of the main ingredients in licorice, GA is consumed as a natural sweetener due to its low toxicity and is also traditionally prescribed for treating asthma, dry cough, and other “pectoral diseases” (Ploeger et al., 2001; Banerjee and Giri, 2016). Through virtual screening and molecular dynamics simulations, multiple studies have found that GA has the potential to bind to multiple essential SARS-CoV-2 proteins (S protein, RBD, 3CLpro, Nsp15, RdRp, ACE2, furin, etc.), which can be used as candidate plant molecules for the treatment of COVID-19 infection (Mahdian et al., 2020; Sinha et al., 2020; Dharmendra Kumar, 2021). Although these molecular autodocking research results may be due to particular molecules’ non-specific interactions, the previous study has shown that GA makes it difficult for the SARS virus to attach and invade target cells and slows down the virus spread from one to another cell. It also has more substantial inhibitory power than ribavirin and other broad-spectrum antiviral drugs in inhibiting the SARS-associated virus’s replication (Cinatl et al., 2003). Furthermore, recent studies have shown that GA potently inhibits the replication of SARS-CoV-2 *in vitro* and relieves the excessive inflammation caused by SARS-CoV-2 in the surrogate mouse model (van de Sand et al., 2021; Zhao et al., 2021).

Based on the above, it seems that GA can be used as the first selective drug to treat COVID-19. But in fact, a high dose of GA is needed clinically to affect virus-infected cells and wipe out the virus (Pilcher, 2003). Nevertheless, we still believe that GA can be a supplement and adjuvant agent to treat COVID-19. This article aims to summarize the immune synergy of glycyrrhizic acid and describe its potential role in reducing COVID-19 complications. Furthermore, this review describes the synergistic phenomenon of glycyrrhizic acid and other drugs, intending to suggest its potential application during COVID-19 treatment.

## Immune System Synergy of GA

The antiviral mechanism of GA against coronaviruses, mentioned before, is mainly on its activity of inhibiting virus replication, preventing virus attachment, or enhancing host cell activity. However, its role in the fight against coronavirus infection is multi-faceted.

Clinical studies have shown that SARS-CoV-2 activates CD4^+^ T lymphocytes and becomes Th1 helper cells after entering the human body. However, most COVID-19 patients have a significant decrease in lymphocytes and T cell subsets, especially CD4^+^ and CD8^+^ T cells (Chen et al., 2020a). The main reasons for this are the innate immune escape mechanism of SARS-CoV-2, and the delayed development of the adaptive immune response, and the prolonged virus clearance time (Grifoni et al., 2020). GA can promote the proliferation of T cells and has Th1 immunological adjuvant activity, thus enhancing the immune system’s resistance to SARS-CoV-2 early (Kim et al., 2013).

When SARS-CoV-2 infects T lymphocytes, it will remain latent in the infected host cells for 12–36 h (Bar-On et al., 2020; Diao et al., 2020). It has been reported that GA could induce apoptosis of host cells in G1 cell cycle arrest and latent virus infection (Cohen, 2005; Curreli et al., 2005), indicating that the roles of GA on apoptosis during the treatment of SARS-CoV-2 is worthy of further investigation.

Except for participating in the immune response, Th1 may secrete granulocyte-macrophage colony-stimulating factor (GM-CSF), which leads to the appearance of inflammatory factors CD14 and CD16 with high IL6 expression and accelerates the development of pneumonia (Tang et al., 2020). The activation of IL-6 is composed of multiple pathways, one of which cannot be ignored is that SARS-CoV-2 infection may activate nuclear factor-κB (NF-κB), and overactivation of NF-κB activates IL-6 amplifier (IL-6 Amp). Subsequently, IL-6 Amp induces various pro-inflammatory cytokines and chemokines, including IL-6, and ultimately enhances IL-6 Amp through positive feedback regulation (Hirano and Murakami, 2020). Inhibition of NF-κB could block the inflammatory response induced by a coronavirus and increase patient survival rate (Dediego et al., 2014b; Yang et al., 2017a). In addition, SARS-CoV-2 may bind to LPS to enhance NF-κB and cytokine responses and promote the development of inflammation and ARDS (Van Gucht et al., 2006; Petruk et al., 2021). GA has anti-inflammatory effects of inhibiting the NF-κB expression (Wang et al., 2011) and significantly inhibiting the production of a variety of cytokines secreted by macrophages, including IL-6 production (Liu et al., 2014).

High mobility group box-1 (HMGB1), as a biomarker of acute lung injury (Qu et al., 2019) and a key mediator of fatal systemic inflammation, could induce cell death (pyroptosis) and immunosuppression, thereby impairing the body’s ability to eradicate microbial infections and leading to death. It could also regulate autophagy and participate in the invasion and replication of SARS-CoV-2 (Street, 2020). Inhibiting the activity of HMGB1 or reducing its release could prevent fatal endotoxemia and sepsis (Bailly and Vergoten, 2020). GA can bind to HMGB1 to disrupt its protein activity, inhibit inflammation, and reduce acute respiratory distress syndrome (ARDS) through the HMGB1-TLR4 signaling pathway (Cohen, 2005).

## The Role of GA in COVID-19 Comorbidities

Clinical investigations have shown that patients with diabetes have an increased risk of adverse consequences after being infected with COVID-19 (Eastin and Eastin, 2020). Obesity has been proven to be another critical risk factor for COVID-19 (Muscogiuri et al., 2020). Lipid raft is a cholesterol-rich microdomain on the cell's plasma membrane, which can be used as an attachment object by the coronavirus and assist it in invading host cells (Lu et al., 2008; Wang et al., 2008; Fantini et al., 2020). Diabetes can cause cholesterol to be loaded into tissues rich in macrophages, which may be one reason for the poor prognosis of COVID-19 patients with diabetes (Wang et al., 2020). When the cholesterol content in the lipid raft decreases, coronavirus invasion (including SARS-CoV-2) will be blocked (Guo et al., 2017; Wang, et al., 2020). There is evidence that GA can reduce the invasion of SARS-CoV-2 by down-regulating the size of the lipid raft domain (Sakamoto et al., 2013; Sakamoto et al., 2015; Baglivo et al., 2020).

In addition to diabetic patients, in some clinical investigations, hypertension is another risk factor that can lead to severe or fatal COVID-19 (Lippi et al., 2020). Patients with hypertension are often accompanied by hypercholesterolemia, and the accumulation of cholesterol in the vascular endothelium can lead to the formation of blood clots or thrombus (Rafieian-Kopaei et al., 2014; Ivanovic and Tadic, 2015). Cholesterol can help SARS-CoV-2 invade host cells, and the latter can promote platelet aggregation and thrombus formation. It may be one of the reasons for the poor prognosis of patients with COVID-19 with hypertension (Manne et al., 2020). There is evidence that GA can reduce the vascular endothelial cell membrane's cholesterol domain while inhibiting platelet aggregation and thrombus formation (Francischetti et al., 1997; Mendes-Silva et al., 2003; Mason et al., 2016). However, it cannot lower blood pressure and can lead to pseudoaldosteronism and hypertension by long-term treatment. The above symptoms can be reduced after treatment with spironolactone (Omar et al., 2012).

Metabolic syndrome (MetS), a clinical syndrome consists of obesity, hyperglycemia, hypertension, and dyslipidemia, is a risk factor for the development of severe COVID-19 and acute respiratory distress syndrome (ARDS) (Felsenstein et al., 2020). The lipopolysaccharide (LPS) in the blood often stays in a high level of MetS patients (Vors et al., 2015; Awoyemi et al., 2018). When SARS-CoV-2 is combined with LPS, it can enhance nuclear factor-κB (NF-κB) and cytokine response, promote inflammation, and develop ARDS (Van Gucht et al., 2006; Petruk et al., 2021). GA can inhibit NF-κB in regulating the inflammatory response induced by LPS (Yu et al., 2005; Wang et al., 2011; Zhao et al., 2016a).

Patients with COVID-19 often have gastrointestinal symptoms, and experiments have shown that SARS-CoV-2 can infect intestinal epithelial cells (Lamers et al., 2020). Clinical studies have shown that patients with inflammatory bowel disease (IBD) infected with COVID-19 can lead to poor recovery, so anti-TNF-α preparations are recommended (Tursi et al., 2020). GA can inhibit TNF activity, reduce intestinal inflammation, and improve IBD (Yang et al., 2017b; Zeeshan et al., 2019).

Brain nerve damage caused by SARS-CoV-2 infection seems very common and has nothing to do with the severity of COVID-19 (Helms et al., 2020). GA’s anti-apoptotic mechanism can adjust the ratio of mitochondrial Bax/Bcl-2 family, affect PI3K/Akt signaling and inhibit HMGB1 activity, resulting in a powerful neuroprotective effect (Kao et al., 2009). Furthermore, GA has been proven to exert powerful neuroprotective properties in neuroinflammation and ischemic brain damage (Kim et al., 2012; Liu et al., 2019).

Accompanying pain in COVID-19 patients is a relatively common phenomenon (Huang et al., 2020). Previous studies have shown that SARS-CoV can activate/increase the expression of activating transcription factor 2 (ATF2) (Dediego et al., 2014a). ATF2 not only can activate pro-inflammatory genes but also play an active role in regulating inflammatory pain. Inhibiting ATF2 can exert not only anti-inflammatory activity but also reduce inflammatory pain (Reimold et al., 2001; Fang et al., 2013). GA was reported likely to inhibit the activity of ATF2 by inhibiting the expression of P38 upstream of ATF2 (Zhao et al., 2016b; Wang and Du, 2016).

## Combination of GA and Other Drugs

Therapeutic drugs for COVID-19 mainly include antiviral agents (remdesivir, ribavirin, hydroxychlo-roquine, chloroquine, etc.) and supporting agents (nitric oxide, sebaceous steroid, etc.) (Li and De Clercq, 2020; Wu et al., 2020). It has been shown that corticosteroid dexamethasone could reduce the mortality of patients with severe COVID-19 by limiting the destructive effects of cytokines. However, dexamethasone had also been reported to suppress the body’s immune system and lead to an increase in plasma viral load (Ledford, 2020; Theoharides and Conti, 2020). We noted that GA could affect the metabolism of steroids and increase its plasma concentration by inhibiting glucocorticoid metabolism (Chen et al., 1991). The combination of dexamethasone and GA effectively reduced the severity of shock in animal experiments (Yu et al., 2005). These data suggested that the combination of dexamethasone and GA could be a promising treatment strategy for patients with COVID-19.

There is a bi-directional relationship between diabetes and COVID-19. Not only patients with diabetes are more susceptible to SARS-CoV-2 infection, but also the prognosis is worse (Apicella et al., 2020; Rubino et al., 2020). Moreover, surveys show that 14% of recovered patients from COVID-19 will have new-onset diabetes (Sathish et al., 2021). One reason for this is that SARS-CoV-2 might directly or indirectly damage pancreatic islets and causes acute β-cell dysfunction followed by type II diabetes (Fignani et al., 2020; Mukherjee et al., 2020; Müller et al., 2021). Another reason is that COVID-19 therapeutic drugs can induce diabetes. For example, some antiviral agents can cause autoimmune damage to pancreatic islet cells. Therefore, after taking the drug, it will cause insulin resistance or abnormal insulin secretion. Ultimately, these antiviral agents will have a greater probability of inducing the occurrence of secondary diabetes (Nakamura et al., 2011). On the other hand, corticosteroids are the culprit of ketoacidosis during the treatment of COVID-19, and their chronic or high-dose use can eventually lead to the onset of diabetes (Alessi et al., 2020; Zhang and Zhang, 2020). Previous studies have shown that GA has an anti-diabetic activity that can improve drug-induced diabetes (Takii et al., 2001; Sen et al., 2011; Yang et al., 2020a). Based on these factors, we recommend that the combined use of GA in the treatment of COVID-19 can effectively improve or even inhibit the occurrence of diabetes.

As a rescue therapy, inhaled nitric oxide (NO) therapy has been used to improve the ARDS caused by COVID-19 (Kobayashi and Murata, 2020). NO could inhibit the replication of coronavirus and reduce lung injury mediated by inflammatory cells and effectively and selectively relax pulmonary blood vessels. These effects of NO could further reduce pulmonary vascular resistance, reduce alveolar cavity edema, and ultimately alleviate ARDS (Åkerström et al., 2005; Martel et al., 2020). It is possible that GA could stimulate and enhance the production of NO by macrophages through up-regulating the expression of the inducible NO synthase (iNOS) gene (Yi et al., 1996; Jeong and Kim, 2002).

Additionally, most of the drugs used in the clinic in treating COVID-19 patients may cause abnormal liver functions (Lai et al., 2020a; Wiersinga et al., 2020). GA has also been used as a hepatoprotective drug with minimal adverse effects, indicating that GA might be suitable for use in combination with various types of drugs to enhance their effects and reduce adverse effects (Cohen, 2005; Lee et al., 2007; Li et al., 2014). Therefore, the effects of GA on liver protection may play a critical role in cooperating with the drug treatment of COVID-19.

## Conclusion

**FIGURE 2:**
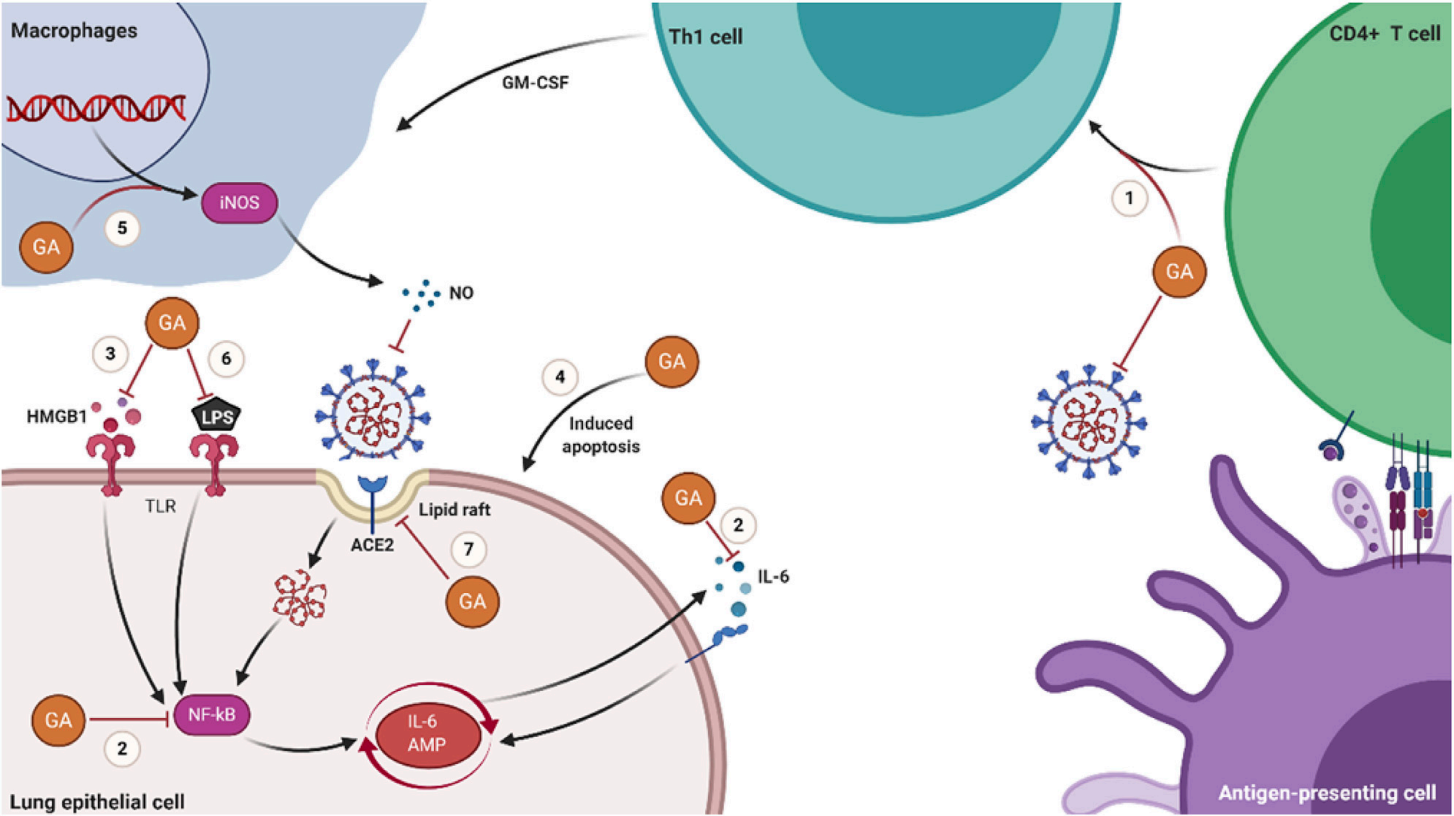
Schematic diagram of the molecular mechanisms of GA in treating COVID-19. (1) GA promotes the proliferation of T cells and has Th1 cell immune adjuvant activity. (2) GA inhibits the production of IL-6 and the activation of NF-κB. (3) GA inhibits the activity of HMGB1 and the signal transduction of the HMGB1-TLR4 pathway. (4) GA induces the apoptosis of host cells latent with SARS-CoV-2. (5) GA stimulates macrophages to produce NO. (6) GA inhibits the inflammatory response induced by LPS. (7) GA prevents the invasion of SARS-CoV-2 by reducing the domain of lipid rafts.

GA is a well-established botanical medicinal molecule used for a long time for antiviral and anti-inflammatory treatments. It has been proved that it can effectively inhibit the invasion and replication of SARS. It has been predicted that it can be combined with multiple proteins of SARS-CoV-2 in molecular docking studies. Moreover, it can also inhibit the replication of SARS-CoV-2 and the release of inflammatory factors *in vitro* and animal experiments. However, GA has been disregarded due to the large dose required as an independent antiviral drug. In this review, we have analyzed the mechanisms of action of GA in the following aspects and illustrated them in Figure 2.

• GA has adjuvant immune activity, which assists the body in the immune response to viruses in the incubation period.
• The anti-inflammatory activity of GA could reduce the cytokine storm caused by the virus.
• GA can reduce or prevent the invasion of SARS-CoV-2 by regulating steroid metabolism.

In conclusion, we believe that the role of GA in the comorbidities of COVID-19 is still worthy of attention. In particular, GA can be used in combination with various drugs to produce a synergistic effect and effectively reduce the mortality of virus-infected patients. Therefore, we regard that the compound can be used as a conventional adjuvant agent to treat COVID-19.
